# Development and validation of the medical professionals resilience scale

**DOI:** 10.1186/s12913-021-06542-w

**Published:** 2021-05-21

**Authors:** Mardhati Ab Rahman, Muhamad Saiful Bahri Yusoff, Nurhanis Syazni Roslan, Jamilah Al-Muhammady Mohammad, Anisa Ahmad

**Affiliations:** Department of Medical Education, School of Medical Sciences, Universiti Sains Malaysia, Health Campus, Kelantan 16150 Kota Bharu, Malaysia

**Keywords:** Resilience, Measurement Scale, Medical Professionals, Medical Education, Medical Doctors

## Abstract

**Purpose:**

Most of the resilience scales were developed for the non-medical population, therefore the purpose of this study was developing and validating a resilience scale for medical professionals namely Medical Professionals Resilience Scale (MeRS).

**Methods:**

A questionnaire development and validation study was conducted. The resilience domains and items were identified and generated through a literature review. The content validation was carried out by content experts and the content validity index (CVI) was calculated. The face validation was performed by medical officers and the face validity index (FVI) was calculated. The final MeRS was administered to 167 medical officers, exploratory factor analysis (EFA) and reliability analysis were performed to assess MeRSs factorial structure and internal consistency.

**Results:**

Four domains with 89 items of medical professionals resilience were developed. Following that, the content and face validation was conducted, and a total of 41-items remained for construct validation. EFA extracted four factors, namely growth, control, involvement, and resourceful, with a total of 37 items. The items CVI and FVI values were more than 0.80. The final MeRSs items had factor loading values ranged from 0.41 to 0.76, and the Cronbachs alpha values of the resilience domains ranged from 0.72 to 0.89.

**Conclusions:**

MeRS is a promising scale for measuring medical professionals resilience as it showed good psychometric properties. This study provided validity evidence in terms of content, response process, and internal structure that supported the validity of MeRS in the measurement of resilience domains among medical professionals.

**Supplementary Information:**

The online version contains supplementary material available at 10.1186/s12913-021-06542-w.

## Introduction

Local and international evidence consistently suggests that physicians are among the front line of healthcare have the highest prevalence of burnout [1,3]. With increasing demands from the complexity of medical practice and eroding public trust, there is now increasing work pressure for physicians which puts them in the vulnerable position. Therefore, resilience seems vital for physicians to survive in this turbulent professional field [4]. Several studies have shown that the presence of resilience among medical professionals would yield positive impacts on both physicians and patients [5, 6].

Generally, resilience is the ability of individuals to cope and deal with challenging or difficult situations while maintaining and improving their wellbeing [7]. However, It has been shown the resilience dimensions are influenced by context and culture [8,11]. For instance, a meta-analysis revealed the lack of a unified view and consensus in resilience [12], while a meta-synthesis demonstrated that 21 resilience scales were developed measuring different sets of resilience domains [7]. In medical professionals context, several qualitative studies that looked into resilience definition from different disciplines proposed that medical professionals resilience refers to a dynamic process where one is driven by a clear sense of purpose, can strike a work-life balance, practice reflection by utilizing adaptive coping and bounce back in the face of adversities, has good interpersonal skills with healthcare members and nurturing work culture [13,17]. Despite the variability in resilience definitions and concepts, it appears that there is less need for a universal definition of resilience. Instead, further research is needed to explore resilience in the specific population context and culture [18].

Although several scales have been developed for measuring resilience, they are not widely adopted and no scale is preferable over the others [7, 19]. Consequently, researchers have little evidence to inform their choice of resilience measurement and may make an arbitrary and inappropriate selection for the population and context. There is a limited number of reviews on the psychometric of resilience scales, and the earliest review was performed to compare the psychometric properties and appropriateness of six resilience scales in the adolescent context [20]. Followed by a more recent review by Windle et al. (2011), in which 15 resilience scales were evaluated against strong quality criteria to assess validity and reliability, however, the conceptual domains of resilience were not adequately addressed [19]. The latest review on resilience scales revealed the most common resilience scales used in healthcare professions were the Brief Resilient Coping Scale (BRCS) and Connor-Davidson Resilience Scale (CD-RISC) [7]. However, it is interesting to note that out of 21 resilience scales reported in the literature, none was developed specifically for medical professionals [7].

Due to the scarcity of literature about the development and validation of resilience scales for medical professionals [7, 21,23], thus it has become a critical need to develop and validate such scale. The primary aim of this study was to develop and validate a scale to measure resilience among medical professionals namely Medical Professionals Resilience Scale (MeRS).

## Methods

**Fig. 1:**
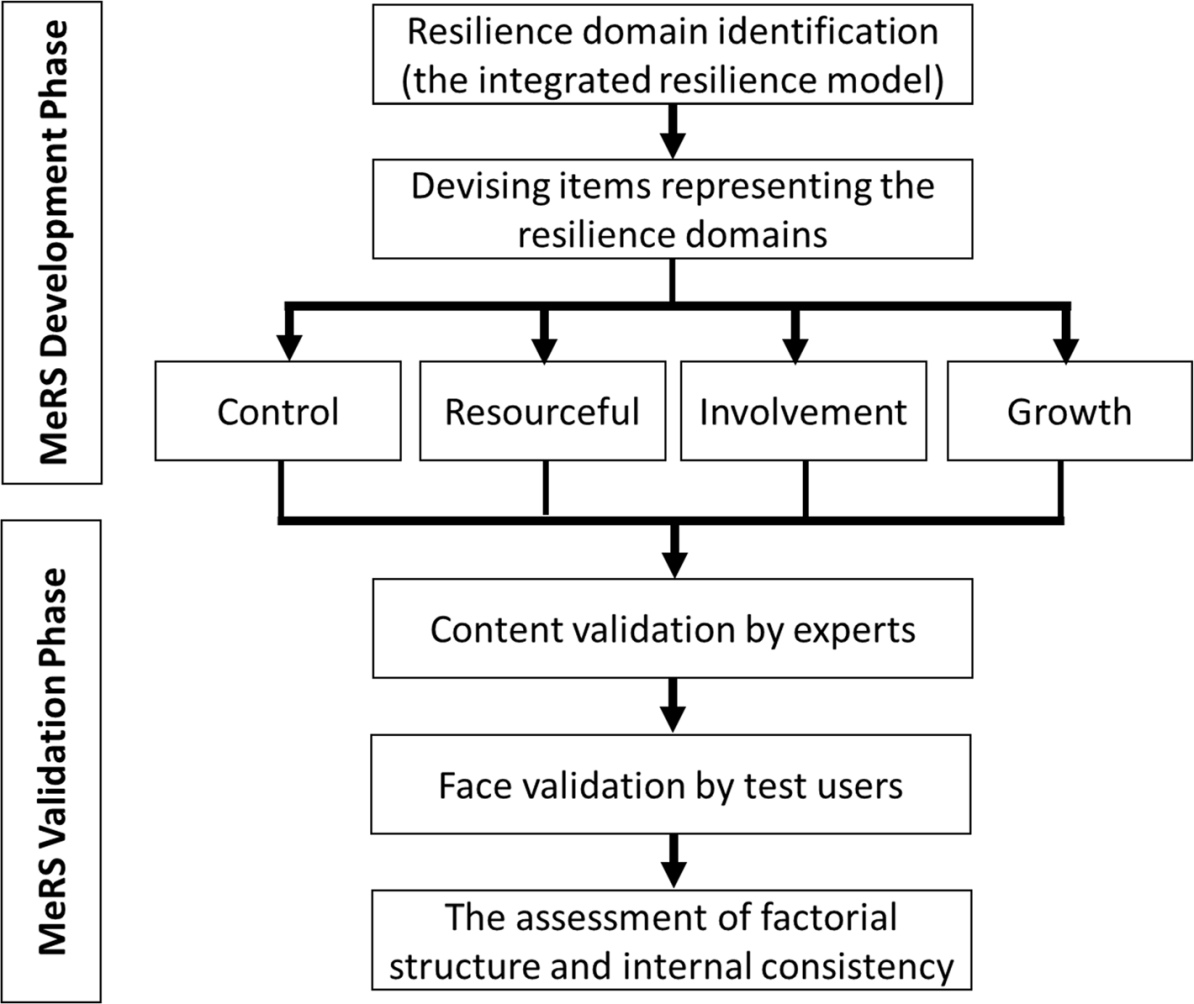
The flow chart for the development and validation process

This study was conducted in two phases. The first phase identified domains of resilience in medical professionals context and was followed by the generation of items according to the identified resilience domains. The second phase validated the resilience domains and items in terms of their content (content validation) and response process (face validation), and later followed by the assessment of factorial structure and internal consistency. Content validity refers to the extent of an instrument is relevant to and representative of the measured construct for a particular purpose [24]. Face validity is defined as the extent of test respondents understanding and interpretation of the test content for a particular purpose [25]. The development and validation process of MeRS are summarised in Fig.1.

### Development of MeRS items

The domains of resilience in medical professionals were established based on the integrated resilience model [7]. The integrated resilience model has the following four domains:

i. ***Control***: Being composed and controlled under stressful adversity;
ii. ***Resourceful***: Being able to find appropriate solutions from available resources to deal with adversity;
iii. ***Involvement***: Being committed to deal with the adversity; and.
iv. ***Growth***: Keep growing and bouncing back stronger from the adversity.

Based on the four resilience domains, items were developed by the researchers through literature review and two research brainstorming sessions that involved all researchers (MAR, MSBY, NSR, JAMM, AA). The items were constructed in the English language representing the resilience domains.

### Validation of MeRS domains and items

#### Content validation

The first version of MeRS (MeRS 1.0) was sent out to a panel of content experts to review the relevancy of MeRS items to the resilience domains. The experts were selected based on their qualification and research experience related to resilience and the minimum number of experts for content validation was set at least six experts [24].

A total of 14 experts were invited to perform the content validation task through an official invitation letter. The experts were provided with a content validation form and requested to judge the degree of relevance of each item to the measured domain based on the recommended 4-rating scales for the content validation (1=the item is not relevant to the measured domain; 2=the item is somewhat relevant to the measured domain; 3=the item is quite relevant to the measured domain, and 4=the item is highly relevant to the measured domain) [24, 26]. They were also requested to provide written comment on any items that were required modifications or subjected to removal. Content validity index (CVI) was calculated based on the following parameters [24, 26].

i. ***I-CVI*** (item-level content validity index): The proportion of content experts giving an item a relevance rating of 3 or 4.
ii. ***S-CVI*** (scale-level content validity index): The average of the I-CVI scores for all items on a scale (i.e., a resilience domain).

The acceptable I-CVI value was set at a minimum of 0.78, while the acceptable S-CVI value was at a minimum of 0.80 [24, 26]. Based on the I-CVI values, items with I-CVI less than 0.80 were rejected, items with I-CVI at least 0.83 but less than 1 were re-discussed, and items with I-CVI of 1.0 were accepted.

#### Face Validation

The face validation was performed after the content validation and the main purpose was to examine the clarity of instructions and language whether there were ambiguities or multiple ways to interpret the items [25]. The second version of MeRS (MeRS 2.0) was sent out to a panel of test respondents to review the clarity of MeRS items. The number of test respondents for face validation was set at a minimum of 10 [25].

In this study, the test respondents were medical officers working in a tertiary hospital. A total of 18 medical officers were invited to perform the face validation task through an online Google Form. They were requested to judge the clarity of each item based on the recommended 4-rating scales (1=item is not clear; 2=item is somewhat clear; 3=item is quite clear and; 4=item is highly clear) [25]. They were also requested to provide written comments on any items that were required modifications. Face validity index (CVI) was calculated based on the following parameters [25]:

i ***I-FVI*** (item-level face validity index): The proportion of test respondents giving an item a clarity rating of 3 or 4.
ii ***S-CVI*** (scale-level face validity index): The average of the I-FVI scores for all items on a scale (i.e., a resilience domain).

The acceptable I-FVI value was set at a minimum of 0.80, while the acceptable S-FVI value was at a minimum of 0.83 [25]. Based on the I-FVI values, items with I-CVI less than 0.80 were rejected, and items with I-CVI more than 0.80 were accepted.

#### The assessment of factorial structure and internal consistency

Following the content and face validation, the third version of MeRS items (MeRS 3.0) was administered to a total of 167 medical officers via a cross-sectional survey to examine the factorial structure and internal consistency. The medical officers were requested to provide rating for each item based on the 4-point Likert scales (1=Strongly disagree, 2=Disagree, 3=Agree, 4=Strongly agree). The MeRS survey form was disseminated to the medical officers through several face-to-face sessions. Informed consent was sought from them before they were given the MeRS survey and their participation in this study was voluntary.

The factorial structure was analyzed by Exploratory Factor Analysis (EFA) using Statistical Package for Social Sciences version 24 (SPSS 24) to assess the number of resilience domains that can be extracted from the MeRS items [27]. The Kaiser-Meyer-Olki (KMO) and Bartletts Test provide parameters to indicate the sampling adequacy and appropriateness of factor analysis. The KMO value of more than 0.7 is considered as a good level of factor distinction based on an adequate sample, and the significant Bartletts Test (p-value less than 0.05) indicates the factor analysis is appropriate (Field, 2009). If the value of KMO value is less than 0.5, researchers should consider collecting more samples [27]. The principal component extraction with varimax rotation was performed to optimize the loading of items to the extracted factors. The minimum factor loading values was set at 0.40 and any items with factor loading less than 0.4 were removed [28].

Following EFA, the reliability analysis was performed to assess the internal consistency of MeRS items, in this study the reliability was represented by Cronbachs alpha coefficient. Field (2009) suggested any scales with Cronbachs alpha values of more than 0.70 are considered as having a high level of internal consistency.

## Results

### The domains and items of MeRS

A total of initial 112 items in the English language were generated from a literature review and these items were grouped into control, resourceful, growth, and involvement domains. During the research meeting, the items were reviewed by all researchers based on the TOP (trait, outcome, process) approach, and a consensus was reached for 89 items of MeRS 1.0 representing the four resilience domains. The final definition of the resilience domain and the total number of items representing each domain are summarized in Table1. These items are rated against a four Likert scale; 1 (strongly disagree), 2 (disagree), 3 (agree), and 4 (strongly agree).

**Table 1:** The final definition of resilience domains and the total items representing the domains

Resilience domain	Definition	Number of items^a^
Control	The quality of being composed and controlled in adversities, and is influenced by the present state inside a person	37 items
Involvement	Being committed to deal with the adversities, and is influenced by the present state externally	26 items
Resourceful	The ability to find appropriate solutions from available resources to deal with adversities, and is oriented for future state externally	19 items
Growth	Being able to grow stronger from adversity, and is oriented for future state internally	7 items

^a^The full list of 89 items can be referred to the supplementary file A

### Content validity index

14 expert panels from various related specialties were invited for the content validation study. Six expert panels accepted the invitation and returned the completed content validation form. The expert panels comprised of two psychiatrists, two medical educationists, one clinical psychologist, and one community counselor. Out of six experts, four females and two males, all of them have worked as an expert in the field for more than 10 years, three Professional Master holders and three Ph.D holders.

The S-CVI score was 0.84. Out of 89 items, 36 were accepted without modification, 26 were rejected (I-CVI of less than 0.80), and 27 were reviewed by the researchers based on the expert panels qualitative feedback. The researchers reached a consensus to revised 17 items and deleted 10 items due to, redundancy or ambiguity. This eventually created MeRS 2.0 with a total of 53 items.

### Face validity index

18 medical officers were invited through mailing invitations. 10 medical officers participated in the face validation study. Based on the rating, the S-FVI was 0.82. 12 items with I-FVI of less than 0.80 were removed and MeRS 3.0 was created with a total of 41 items.

### Internal Structure Assessment through Exploratory Factor Analysis

Out of 167 invited medical officers, 160 returned the questionnaires with a response rate of 95.8%. Most participants were 31 to 35 years old (75.6%), female (50.6%), Malay (68.1%), married (71.9%), were in medical-based specialties (54.4%), and had a clinical experience of more than five years (90.6%). The data was found to have a normal distribution, a significant Bartletts test, and a Kaiser-Mayer-Olkin (KMO) value of 0.84, suggesting excellent sampling adequacy.

EFA was conducted on all 41 items using Principal Axis Factoring (PAF). Initial EFA analysis suggested a total of 12 factors may be extracted based on eigenvalue of 1 as per Kaisers criteria. Guided by the domain identification stage and the scree plot, only the first four factors with the highest eigenvalues were rotated through the force extraction. 37 items were found to have acceptable factor loadings from 0.407 to 0.764. Four items were removed due to factor loading was less than 0.40; *I never give up in any situation*, *I know my limit when working on challenging task*, *I find different ways to handle difficult situations* and *I find it worth to do same things over again*.

As the items that load highly on factor 1 were related to the future state and internal conditions, factor 1 was named as the growth domain. The items that loaded on factor 2 all were influenced by the present state and internal conditions, hence named as the control domain. The items that loaded on factor 3 were influenced by the present state and external condition, hence labeled as the involvement domain. Finally, all four items that loaded on factor 4 were related to the ability to find appropriate solutions from available resources and named as the resourceful domain.

### Reliability Analysis

The final 37 items were found to have a Cronbachs coefficient alpha of 0.914 (scale level). The Cronbachs coefficient alpha for the involvement domain was 0.719, the resourceful domain was 0.779, the control domain was 0.818, and the growth domain was 0.891.

### Scoring of MeRS

Similar to other resilience instruments, a higher score of MeRS indicates a greater resilience and vice versa. There was no reverse scoring as there were no negative items. Three levels of resilience competency (exceptional, established, and developing) were established based on the highest 27% and the lowest 27% score respectively [29] as shown in Table2.

**Table 2:** The total score for MeRS with a corresponding level of resilience competency

MeRS domain (n item)	Level of resilience competency (total score)		
	Developing (low)	Established (moderate)	Exceptional (high)
Growth (15)	1527	2847	4860
Control (12)	1221	2238	3948
Involvement (6)	611	1218	1924
Resourceful (4)	47	812	1316
Global score (37)	3766	67118	119148

The detail I-CVI, S-CVI, I-FVI, S-CVI, and factor loadings of 37 items as well as the resilience domains internal consistency are summarized in Table3.

**Table 3:** The I-CVI, S-CVI, I-FVI, S-FVI, Factor Loading and Reliability of the Four Domains of MeRS (Final Model)

Domains	Items	I-CVI	S-CVI	I-FVI	S-FVI	Factor Loading	Cronbachs Alpha	Eigenvalue (rotated)	Total % of variance
**Growth**	I can succeed if I keep trying	1.00	0.95	0.90	0.93	0.726	0.89	6.138	14.97
	I believe everything happens for a reason	1.00		0.90		0.672			
	When I face new situations, I will learn from it	1.00		1.00		0.640			
	I believe there is a wisdom behind everything in life	1.00		0.90		0.638			
	I believe every problem comes with a solution	0.83		1.00		0.635			
	I seek help to achieve my goals if necessary	0.83		1.00		0.584			
	I believe by helping others, I am helping myself too	1.00		0.90		0.576			
	I have goals to achieve	1.00		0.90		0.569			
	I believe good planning is a key to success	1.00		0.90		0.565			
	I believe self-motivation will change the final outcome	0.83		0.90		0.561			
	I believe hard work really pays off in the end	0.80		1.00		0.526			
	I am aware of my strengths and abilities	1.00		1.00		0.475			
	I am positive I will be successful in the future	1.00		0.90		0.465			
	When my work is criticized, I cope positively by trying harder the next time	1.00		0.90		0.448			
	Believing in myself helps me to face any difficulties	1.00		0.90		0.423			
**Control**	I am firm with my stand	1.00	0.93	0.80	0.89	0.628	0.818	4.679	11.41
	I can adapt to change at work situations	1.00		1.00		0.604			
	People always believe in me to make difficult decision	0.83		0.70		0.586			
	I am comfortable working in new environment	1.00		0.80		0.548			
	I spend my life doing something great	0.83		0.80		0.501			
	I have good coping skills when dealing with stress	0.80		0.90		0.491			
	I am proud of my own accomplishments	0.83		0.80		0.489			
	I become a stronger person when facing difficulties at work	1.00		1.00		0.478			
	I always give my best at work	1.00		1.00		0.472			
	I feel energetic doing my work even in difficult situations	0.83		1.00		0.459			
	I can maintain interest in my work	1.00		0.90		0.419			
	My colleagues can always rely on me	1.00		1.00		0.407			
**Involvement**	I can stay calm in hard situations	1.00	0.92	0.90	0.92	0.751	0.719	3.279	8.00
	I can handle unpleasant feelings	0.83		0.80		0.724			
	I always try to stay calm in any situation	1.00		0.90		0.564			
	I can control my anger	1.00		1.00		0.544			
	I am in control of my surroundings	0.83		0.90		0.504			
	I am good at adapting myself to different situations	0.83		1.00		0.461			
**Resourceful**	I know who to talk to when I have a problem	1.00	1.00	0.90	0.90	0.764	0.779	3.152	7.69
	I know where to go if I need help	1.00		1.00		0.738			
	I always have someone by my side when I have problems	1.00		0.80		0.702			
	I figure out ways to solve my problems by talking about them	1.00		0.90		0.683			

*I-CVI* item-level content validity index; *S-CVI* scale-level content validity index; *I-FVI* item-level face validity index; *S-FVI* scale-level face validity index

## Discussion

This study contributed several important scientific outcomes related to the resilience measurement in the medical context. First, this study provided four pieces of evidence (i.e., content, response process, factor structure, and internal consistency) to support the validity of a new resilience scale for medical professionals. Second, the 37-item MeRS (MeRS-37) demonstrated an acceptable level of content validity as all items had CVI more than 0.80. Third, the MeRS-37 demonstrated an acceptable level of response process validity as all items have FVI more than 0.80. Fourth, the items of MeRS-37 had nicely loaded into four factors (i.e., subscales) namely control, resourceful, involvement, and growth. Finally, the MeRS-37 demonstrated a high level of internal consistency as Cronbachs alpha values of the scale and subscales were more than 0.70. These results indicated the MeRS-37 is a promising scale for measuring the resilience of medical professionals.

This study provided four pieces of evidence to support the validity of MeRS-37 in terms of content, response process, and internal structure represented by factor structure and internal consistency. Generally, validity describes how well one can trust the interpretations of test scores for a specific purpose based on evidence and theory. There are five sources of validity evidence to support the construct validity [30]: (i) content validity describes how well instrument items represent the intended construct, (ii) response process (i.e., face) validity describes the relationship between the intended construct and the thought processes of test-takers, (iii) internal structure validity describes reliability (internal consistency) and factor structure, (iv) relations to other variables describes the correlation of test scores with scores of another test assessing the same construct, and (v) consequences describes how well the test scores make a difference. These are categories of evidence that can be collected to support the construct validity of inferences made from instrument scores. In this study, three categories of validity had been provided to support the validity of MeRS-37. Future studies should be conducted to provide more evidence supporting the MeRS-37 as a valid resilience scale for medical professionals.

The MeRS-37 demonstrated an acceptable level of content and face validity as it had CVI and FVI more than 0.80. The item development was guided by the recommended guidelines [24, 25, 31, 32], literature review, expert opinions, assessment of existing scales [7, 19], and indicators of that domain [33, 34]. All the items were simplified and positively keyed to make MeRS less cognitively demanding. The MeRS items were developed by a thorough literature review and several brainstorming sessions among researchers, that lead to the generation of 89 initial items (MeRS 1.0). The 89 initial items underwent an extensive content validation process that leads to the removal of 36 items, and the main reason due to the items was less relevant to the resilience domain for medical professionals. The refined 53 items of MeRS (MeRS 2.0) underwent the face validation process that leads to further removal of 12 items, and the primary reason due to the lack of comprehensibility. The refined 41 items of MeRS (MeRS 3.0) contained the items with an acceptable level of relevancy and comprehensibility as indicated by CVI and FVI values of more than 0.80 [24, 25]. The 41 items were further scrutinized through factor and reliability analysis that lead to the final version of MeRS (4.0). The content and face validation processes had significantly removed more than 50% of initial items, and hence some of the removed items may represent important attributes of medical professionals resilience. The possible reason for this result could be due to the limited sample that was confined to a single medical center, and the participants were only medical officers from internal medicine and surgery departments. Therefore, further studies should be carried out in different settings and diverse participants from different fields of medicine to verify the content and face validity of MeRS. Nevertheless, this study provides important evidence to support the content and face validity of MeRS in a medical professional setting, and hence it is a potential scale to measure their resilience.

The factor analysis had proposed a final MeRS with 37 items and four factors (i.e., subscales) - namely control, resourceful, involvement, and growth. The item factor loadings to the four subscales were more than 0.40, indicating an acceptable representation of the intended factors [27]. The four subscales corresponded to the integrated resilience model proposed by Wadi et al. (2020) and aligned with the theoretical concept of resilience as a multidimensional construct [35, 36]. The control subscale is the most common construct in 21 resilience scales [7] and studies have postulated a convergence between emotional regulation and the concept of control. For example, the composure domain from the Predictive 6-factor Resilience Scale (P6RS) is primarily about emotional regulation and the ability to recognize, understand, and act on internal prompts and physical signals [37]. Some of the items in the Adolescent Resilience Scale which are I can stay calm in hard circumstances and a negative item *I have difficulty in controlling my anger* represent emotional regulation [38]. This fits well with the control subscale which we describe as being composed and controlled under stressful adversity. In the Resilience in Midlife (RIM) scale, resourcefulness was regarded as the underlying mechanism that comprised resilience and was named as one of the factors: family and social networks. The example of items representing this domain is *Have someone to help me if needed* and *Rely on the family in tough times* [39]. This is primarily relevant to the resourceful subscale which we define as *Being able to find appropriate solutions from available resources to deal with adversity*. Up to now, several existing resilience scales have described involvement as commitment [7]. For example, The Resilience Scale for Adults developed by Friborg et al. (2006) describes involvement in the item *I keep up my daily routines even at difficult times*. This conclusively showed that the concept of involvement described in other resilience scales aligned with our involvement subscale which is described as being committed to deal with adversity. Wadi et al. (2020) reported out of 21 resilience scales, 16 scales have considered growth as one of the factors that constitute resilience. In these 16 scales, the growth subscale was distinctively described as coping, empowerment, goal setting, strengthening the effect of stress and vision. For example, in the Predictive 6-factor resilience scale, the vision was sorted as one of the domains which include the concept of goal setting while in the Youth Resiliency: Assessing Developmental Strengths scale [40] labeled this subset as empowerment. Together these studies provide important insights into the concept of growth which we describe as keep growing and bouncing back stronger from adversity. In short, this study provides essential evidence to support the factor structure of MeRS-37.

Besides, the internal consistency of the MeRS-37 was found to be high as indicated by Cronbachs alpha values of more than 0.70 [27] either at the scale or subscale level. This result demonstrated that MeRS-37 has a good ability to consistently measure the resilience attributes of medical professionals. A review of six resilience scales [20] (Baruth Protective Factors Inventory, ConnorDavidson Resilience Scale, Resilience Scale for Adults, Adolescent Resilience Scale, Brief-Resilient Coping Scale, and Resilience Scale) demonstrated that all the scales reliabilities were assessed using Cronbach alpha values. The Cronbach alpha values of all the six scales ranged from 0.67 to 0.91 with only one scale having a reliability value exceeding 0.90 which was Resilience Scale [20]. Besides, a review had reported that the Cronbachs alpha coefficients of the Resilience Scale ranged from 0.72 to 0.94 supporting its internal consistency [41]. The evidence reviewed here seems to suggest that MeRS has an equal or better internal consistency as compared to existing resilience scales. The MeRS-37 might be a reliable resilience scale for other medical professionals such as house officers and medical specialists or even for medical trainees, as it could be assumed that their resilience attributes are similar to medical officers. These facts further warrant more studies should be conducted to verify the reliability of MeRS-37 in different settings and contexts.

Despite the positive results, this study has several limitations that should be considered for future consideration. First, the MeRS-37 is a self-administered questionnaire and is inherently confined to a single self-assessment [42], thus bias could arise due to the self-enhancement [43]. Thus, the MeRS scores should be triangulated with other sources of evaluation is recommended and this approach will hopefully pay off in the long run [44]. Second, this study only confined to three categories of validity which were content, response process, and internal structure, therefore, future studies could be carried out to investigate other categories of validity which are the relationships of MeRS-37 with other variables and its consequences [30]. The investigation of this validity evidence could be established by correlating the inventory with other scales measuring the same factors for examples, Academic Resilience Scale [45], Resilience Scale [46], while the consequences of MeRS scores could be established by determining a cut-off score that warrants for interventions. The last limitation is the scoring system of MeRS scores was calculated using the discrimination index method by Ebels, therefore it is purely based on a theoretical formula that requires more verification of its transferability to the clinical practices. Despite these limitations, this study had provided essential validity evidence to support the validity of MeRS as a promising resilience scale for medical professionals.

## Conclusions

MeRS is a promising scale for measuring medical professionals resilience as it showed good psychometric properties. This study provided validity evidence in terms of content, response process, and internal structure that supported the validity of MeRS in the measurement of resilience domains among medical professionals. Further validation is required to provide more evidence to support the validity of MeRS. In the future, this study will form the basis to explore resilience from the perspective of other medical professionals such as medical specialists and house officers as well as other healthcare workers such as healthcare trainees, medical assistants, nurses, and attendants. This is in line with the recommendation that defining resilience should take into account the social and contextual factors to generate a more comprehensive understanding .

## Supplementary Information

**Additional file 1.**

## Data Availability

The datasets used and/or analysed during the current study are available from the corresponding author on reasonable request.
