# Supplementary material for: Development and validation of the medical professionals resilience scale
Source: BMC Health Serv Res. 2021 May 21;21:482. doi: 10.1186/s12913-021-06542-w (PMC8139025; doi:10.1186/s12913-021-06542-w)
Supplement: Supplementary file 1 — Additional file 1. [file 12913_2021_6542_MOESM1_ESM.docx]

**SUPPLEMENTARY FILE A**

**Development and Validation of the Medical Professionals Resilience Scale**

Mardhati Ab Rahman (mardhatiabrahman@gmail.com), Muhamad Saiful Bahri Yusoff (msaiful_bahri@usm.my; ORCID: 0000-0002-4969-9217), Nurhanis Syazni Roslan (nurhanis_syazni@usm.my; ORCID: 0000-0002-5833-1529), Jamilah Al-Muhammady Mohammad ([jamilahkb@usm.my](mailto:jamilahkb@usm.my); ORCID: 0000-0002-1541-9504), Anisa Ahmad (anisa@usm.my; ORCID: 0000-0001-8089-0637)

Department of Medical Education, School of Medical Sciences, Universiti Sains Malaysia, Health Campus, 16150 Kota Bharu, Kelantan, Malaysia.

The full list of 89 items by the resilience domains

| **Domain (number of items)** | **Item** |
| --- | --- |
| **Control (37)** | I have difficulty in controlling my emotion* |
|  | My behaviour at work depends on my daily mood* |
|  | I can control my anger |
|  | I can stay calm in hard situations |
|  | I am good at handling unpleasant feelings |
|  | I have difficulty dealing with most problems* |
|  | I am a strong person |
|  | Self-belief usually helps me get through life |
|  | I am proud of my own accomplishment |
|  | I believe there is a wisdom behind everything in life |
|  | I believe everything happens for a reason |
|  | I can easily make friends |
|  | I am good at keeping friends |
|  | I am a good team player |
|  | I can understand others’ facial expression |
|  | I am easily demotivated by criticism* |
|  | I can maintain interest in my work |
|  | I always try to stay calm in any situation |
|  | My sense of humour helps me to overcome difficult times |
|  | Most problems can be solved by just ignoring them* |
|  | I believe good planning is a key to success |
|  | Believing in myself helps me to face any difficulties |
|  | I pray a lot whenever I am in difficult situations |
|  | I take the lead to solve group problems |
|  | I know my limit when working on challenging task |
|  | I work according to the group plan |
|  | I learn from mistakes to become a better person |
|  | I believe being patience is good for my work |
|  | Failure to deal with difficult situation leads to negative consequences |
|  | I believe the harder I try, the more I can achieve |
|  | I believe no one can do harm to me |
|  | What I do today determine what happens to me tomorrow |
|  | I am in control over what happens to me |
|  | My fate will not change regardless of my effort* |
|  | I am good at changing other’s thought on something |
|  | Most people will pay attention to what I say |
|  | I believe self-motivation will change the final outcome |
| **Resourceful (26)** | I can succeed if I keep trying |
|  | I believe nothing can stop me from reaching my goals |
|  | I can sense if other people are lying |
|  | I can sense other’s mood based on their facial expression |
|  | I think properly before I do something |
|  | My colleagues can always rely on me |
|  | I am good at time management |
|  | I believe I am good in solving my own problems |
|  | I believe every problem comes with a solution |
|  | I never give up in any situation |
|  | I seek help to achieve my goals if necessary |
|  | I am aware of my strengths and abilities |
|  | I like to challenge myself by trying out new things |
|  | I like to explore new things |
|  | I gain valuable experience during difficult times |
|  | I am comfortable doing unfamiliar things |
|  | People always believe in me to make difficult decision |
|  | I am able to handle many things at once |
|  | I am good at adapting myself to different situations |
|  | When I am in difficult situations, I can manage my feelings in different ways |
|  | I know who to talk to when I have a problem |
|  | I figure out ways to solve my problems by talking about them |
|  | I know where to go if I need help |
|  | I find different ways to handle difficult situations |
|  | I believe I have a bright future ahead |
|  | I always have someone by my side when I have problems |
| **Involvement (19)** | I feel energetic doing my daily work |
|  | I believe society gets benefit from my work |
|  | I love daydreaming rather than living in reality* |
|  | I rely on people to decide for me* |
|  | I always give my best at work |
|  | I believe by helping others, I am helping myself too |
|  | I usually work according to my plans |
|  | I spent my life doing something great |
|  | I am comfortable working in new environment |
|  | I always give up when things look hopeless* |
|  | I am in control of my surroundings |
|  | I have a right to my beliefs |
|  | I am firm with my stand |
|  | I believe I can achieve my goals if I work hard |
|  | I am careful in situations that could get me into trouble |
|  | I avoid working with people who could get me into trouble |
|  | I find it worth to do same things over again |
|  | I believe hard work really pays off in the end |
|  | I am the one who gets benefit from my hard work |
| **Growth (7)** | I can adapt to change at work situations |
|  | I become a stronger person when facing difficulties at work |
|  | I have goals to achieve |
|  | When I face new situations, I will learn from it |
|  | When my work is criticized, I cope positively by trying harder the next time |
|  | I have good coping skills when dealing with stress |
|  | I am positive I will be successful in the future |

Note: (*) indicates negative item
